# Comparative Molecular Dynamics Reveals How LRRK2 Inhibitors Distinguish G2019S from Wild-Type

**DOI:** 10.1007/s11064-025-04520-w

**Published:** 2025-08-13

**Authors:** Chuancheng Wei, Choon Han Heh, Lei Cheng Lit, Sek Peng Chin

**Affiliations:** 1 Department of Pharmaceutical Chemistry, Faculty of Pharmacy, Universiti Malaya, Kuala Lumpur, 50603 Malaysia; 2 Department of Physiology, Faculty of Medicine, Universiti Malaya, Kuala Lumpur, 50603 Malaysia

**Keywords:** Molecular dynamics, LRRK2 inhibitors, G2019S/WT selectivity, Kinase-Ligand interaction, Conformational changes

## Abstract

**Abstract:**

Leucine-rich repeat kinase 2 (LRRK2) has become a critical drug target in Parkinson’s disease, with mutation-selective inhibitors offering promising potential for precision medicine. However, the structural similarity between G2019S and wild-type kinases presents a significant challenge in developing selective inhibitors. Although recent advances have led to inhibitors that selectively target G2019S or wild-type kinases, the selectivity mechanism of these inhibitors remains unclear. We employed molecular dynamics simulations to investigate and explore kinase-ligand interactions and identify the underlying mechanisms of selectivity. The results suggest that ligand binding drives the conformational changes, which is a key contributing factor to selectivity, rather than the strength of the ligand binding. The ligand-induced conformational changes lead to kinase destabilisation and inactivation. Additionally, key residues, such as Tyr2018 and Asp2017, were found to play pivotal roles in the selectivity. These insights underscore the importance of incorporating conformational dynamics into the design of future LRRK2 mutant-selective inhibitors.

**Graphical Abstract:**

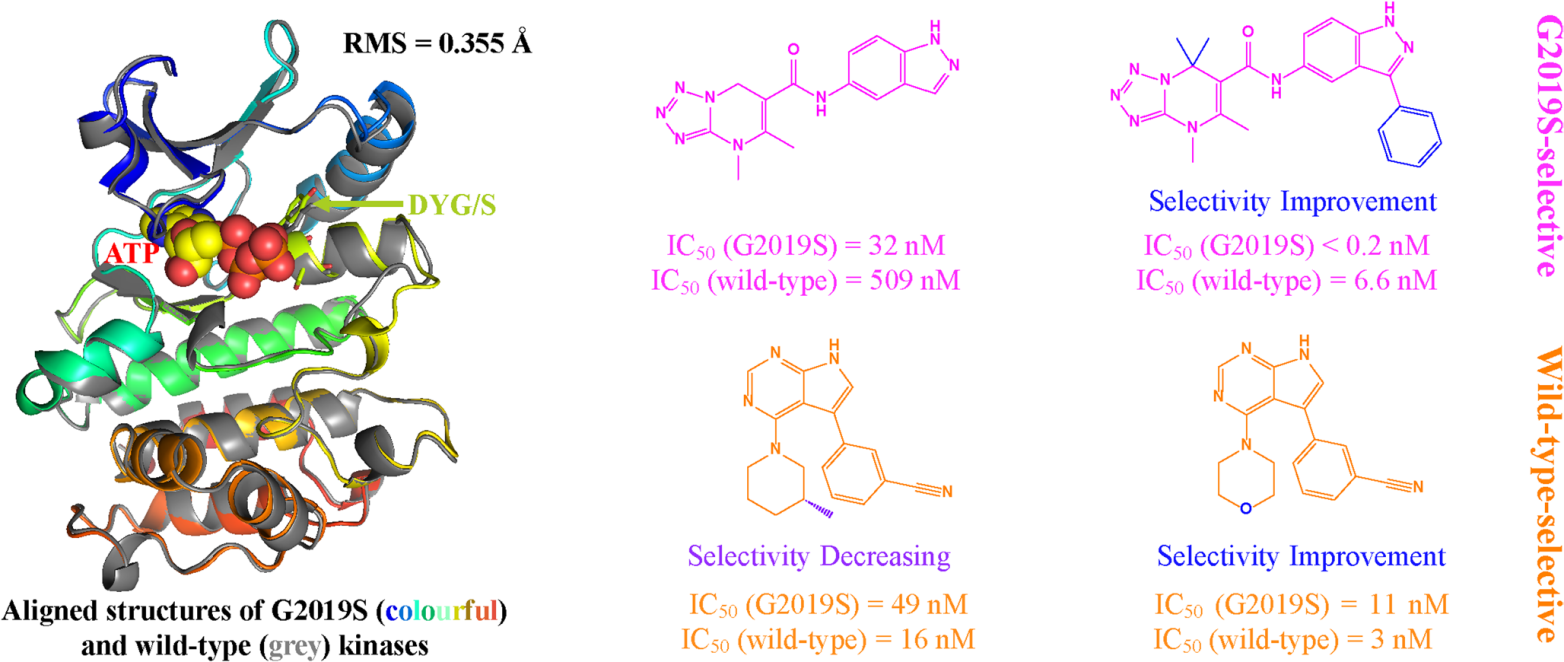

**Supplementary Information:**

The online version contains supplementary material available at 10.1007/s11064-025-04520-w.

## Introduction

As the second most prevalent neurodegenerative disorder, Parkinson’s disease has affected around 1–2% of individuals over 65 years old [1]. Despite extensive efforts, stopping Parkinson’s disease progression has consistently failed in clinical trials, primarily due to the limited efficacy of candidate drugs [2]. This highlights the urgent need for more precise therapeutic strategies. Playing a crucial role in the progression of Parkinson’s disease, leucine-rich repeat kinase 2 (LRRK2) has gained great prominence as a potential drug target [3]. As a multi-domain protein, LRRK2 consists of approximately 2700 residues, folded into seven domains, including WD40, ATPase, and GTPase, which serve as key targets for drug design [4]. The seven domains of the full-length LRRK2 structure [5] mediate different functional processes [4, 6], including monomerisation, dimerisation, and phosphorylation.

Several mutations occurred in various domains, among which one of the most causative mutations, G2019S in the kinase domain, is the most common and strongly associated with increased kinase activity [3, 7, 8] and has attracted the most pharmacological and inhibitor design studies [9]. Compared to non-selective LRRK2 inhibitors, many of which lack selectivity and inhibit both the mutant (G2019S) and wild-type (WT) kinases, G2019S-selective inhibitors offer a promising strategy for precision medicine by minimising off-target effects associated with non-selective LRRK2 inhibition [10]. Clinical trials revealed that non-selective LRRK2 inhibitors targeting mutant and wild-type kinases may cause off-target side effects and lung pathology [11]. However, despite intensive efforts, no G2019S-selective inhibitors have yet entered clinical trials, and the structural basis for their selectivity remains poorly understood [12].

**Fig. 1:**
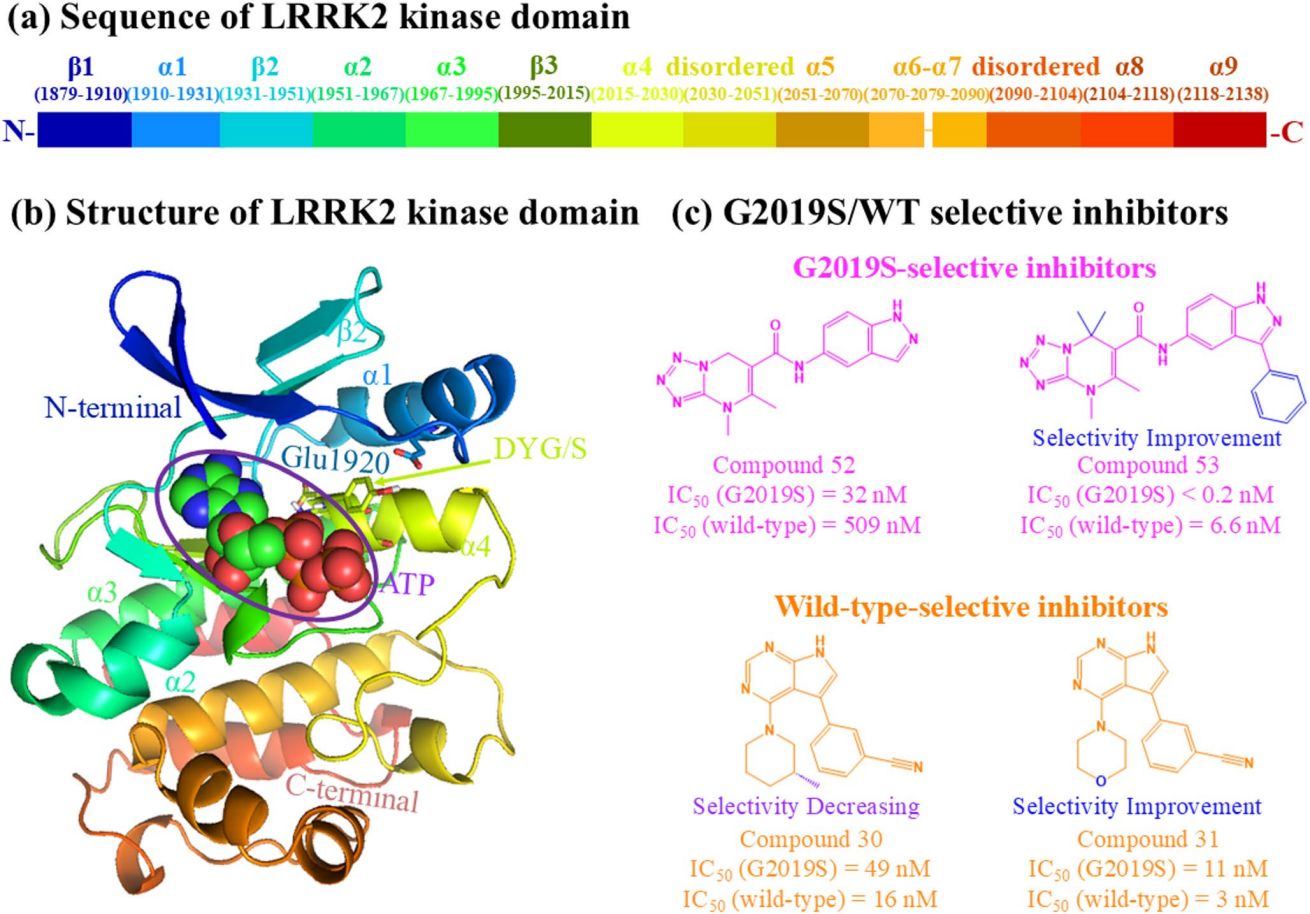
The **a** sequence and **b** structure of the LRRK2 kinase domain with ATP bound (PDB ID: 7LHW). **c** Chemical structures of four G2019S/wild-type selective inhibitors

The kinase domain of LRRK2 consists of fourteen secondary structural segments, including nine α-helixes, three β-sheets, and two disordered regions, as shown in Fig. 1a and b. To describe the conformational changes clearly, we defined the LRRK2 segments using the recently reported LRRK2 full-length monomer structure (PDB ID: 7LHW) [13]. The β1 (containing the G-loop in its terminal regions after Gly1990), α1, β2, α2, α3, and α4 form a pocket for the ATP [14]. Specifically, the DYG/S motif (residues 2017–2019) in the α4, where the G2019S mutation occurs, is an essential part of the activation loop [14, 15]. The rotation of the DYG/S motif controls the pocket volume and modulates kinase activity [14, 15]. Besides, three residues, Asp2017 in α4, Lys1906 in G-loop, and Glu1920 in α1, interact with the γ-phosphate of ATP and Mg²⁺, stabilising the kinase-ATP interactions. Therefore, the separation of Asp2017 and Glu1920 could reduce the phosphorylation activity of the kinase [14, 15]. Positioned adjacent to the kinase pocket, the C-terminal (α5-α9) stabilises the kinase domain’s overall structure and regulates the catalysis [16]. Notably, the high similarity between G2019S and wild-type kinase structures poses a major challenge to achieve the inhibitors’ selectivity via conventional competitive inhibitory mechanisms [17]. Therefore, elucidating the atomic-level mechanisms by which ligands distinguish between the two forms would provide valuable insights to guide the rational design of selective inhibitors.

Nevertheless, several studies have identified inhibitors showing differential selectivity for G2019S and wild-type LRRK2, despite their high structural similarity [18, 19]. In particular, the scaffold of dihydrotetrazolo[1,5-a]pyrimidine was selective for G2019S, and pyrrolic[2,3-b]pyridine was evidently selective for the wild-type [9]. As shown in Fig. 1c, compound 53 (EB-42486) exhibits high selectivity towards the G2019S as indicated by its IC_50_ values [20]. Compound 52 also shows a similar selectivity, but slightly reduced selectivity than compound 53 [20]. Compound 31 (PF-06447475) is another promising candidate, demonstrating pronounced selectivity for the wild-type [21]. Compound 30 has the poorest selectivity, making it the least promising candidate [21]. Yet, a clear explanation of how subtle alterations in chemical structure influence selectivity remains elusive. Therefore, this study employed comparative molecular dynamics simulations to elucidate the structural mechanisms driving the selectivity of these mutant-targeted compounds.

## Methods

### System Preparation

The full-length LRRK2 structures of wild-type (PDB ID: 7LHW) and G2019S (PDB ID: 7LI3) that were retrieved from the Protein Data Bank (PDB) were used in this study. The structures were selected to ensure consistency, as both were resolved under identical experimental conditions within the same study [4]. The kinase domain (residue 1879–2138) was retained [4], while other domains were removed using Discovery Studio 4.5 [22]. The missing residues in the kinase domains were fixed using “Build Homology Models” in the Discovery Studio 4.5 [22].

The 3D conformations of these four selective ligands (Fig. 1c) were generated and minimised under the MM2 force field [23] in Chem3D [24]. Then, the orientations in which these ligands bind to the kinase active site were explored at exhaustiveness 100 using AutoDock vina 1.1.2 [25]. Selected representative docking poses were used as the initial conformations for molecular dynamics simulations, as justified and discussed in the binding mode analysis (Sect. 3.1). A total of 8 systems were simulated: four ligands (compound 53, 52, 30, and 31) in G2019S and wild-type kinases, respectively.

### Equilibration and Production Simulation

The selected complexes from the molecular docking were solvated in TIP3P water at an octahedral radius of 10 Å, and the ions of Na^+^ and Cl^−^ were added to equal the charge using AmberTools23 [26]. The restrained electrostatic potential (RESP) charges of the small molecules were calculated using Gaussian09 [27] at HF/6–311 g(2d, p) level [28, 29], and these molecules were described using the GAFF2 force field [30], while the proteins were defined using the ff19SB force field [31].

For all systems, conjugate gradient and steepest descent were first carried out for 10,000 steps each to minimise the system energy under the constraint for the kinase-ligand complexes, followed by the optimisation of the entire system without constraint. Subsequently, the systems were heated to 310 K using the canonical ensemble (NVT) for 400 ps under a constraint. Finally, in the constant temperature and pressure (NPT) phase under a constraint, the systems were equilibrated for 500 ps.

Based on the recommendation from a previous simulation work of the LRRK2 kinase domain [32], a following 500 ns production molecular dynamics simulation (400 ns for stabilisation and the subsequent 100 ns for sampling) was executed at the cut-off of 10 Å under the NPT. Three parallel runs for the production simulations of 500 ns were performed on all 8 systems under the PMEMD.CUDA [33–35] from AMBER 22 [36].

### Trajectories Analysis and MM/PBSA Calculation

The root-mean standard deviation (RMSD) for the protein and the ligand, root-mean standard fluctuation (RMSF), and the radius of gyration (RoG) for each run were calculated using MDAnalysis [37–39] and visualised using ggplot2 [40]. The last 100 ns of one of the most stable representative trajectories for each system were selected for the following conformational and interactional analysis.

Considering the selectivity of the binding differences may be caused by the conformations of the ligands and the Gly-Ser mutation in residue 2019, the distance between the mass centres of the ligand and residue 2019 in the last 100 ns was calculated using MDAnalysis [37–39]. Combining with the RMSD of the ligand in the last 100 ns, probability distribution was calculated using the ddtpd v1.3 [41] and was visualised into the free energy landscape (FEL) via ggplot2 [40]. The crucial interval of 1 ns (a total of 100 frames) that holds the highest count of frames in the ground minima from the FEL was identified using the Crucial Interval Calculation (CIC) package [42] for all systems. The frame located in the ground minima and nearest to the centre of the crucial interval was extracted using Chimera 1.17.1 [43] and visualised using PyMol 1.8 [13] as the representative structures for binding conformation analysis.

The Molecular Mechanics Generalised Boltzmann Surface Area (MM/GBSA) was calculated on the crucial interval using the MMPBSA.py package [44] in AmberTools 23 [26]. The principal components analysis (PCA) was performed for the entire simulation trajectory using MDAnalysis [37–39], and the principal components for the crucial interval in the free energy landscapes were converted into a dynamics cross-correlation matrix (DCCM) and visualised using ggplot2 [40].

## Results and Discussions

### Binding Modes Predicted by Molecular Docking

The top five docking poses of each ligand on both G2019S and wild-type LRRK2 kinases were analysed (Table S1) to identify the most reliable binding conformation for subsequent molecular dynamics simulations.

For compound 53, distinct binding modes were observed between the two kinases. In G2019S, the dihydrotetrazolo[1,5-a]pyrimidine moiety predominantly points rightward in the top four poses, whereas in pose 5 it adopts a leftward orientation similar to that seen in the wild-type. Conversely, in the wild-type kinase, the moiety points leftward in poses 1, 3, and 4. Notably, pose 5 in the wild-type shows the rightward orientation typically seen in G2019S, and pose 2 adopts an upright orientation. These observations suggest that the G2019S mutation may influence the preferred orientation of compound 53, despite being a single residue substitution.

For compound 30, several docking poses appeared outside the predicted binding pocket (e.g., pose 2 in G2019S, and poses 1, 3, and 5 in wild-type). The G2019S-30 system displayed high variability, with four distinct orientations among the top five poses, making it difficult to define a consistent binding mode. Therefore, only the most plausible pose (pose 1) was retained for further analysis. In contrast, wild-type-30 showed better convergence, with poses 2 and 7 sharing a similar orientation; pose 2 was selected for the next simulations.

For compounds 52 and 31, the top five docking poses were largely consistent, with more than three poses closely resembling pose 1. This indicates a well-defined binding mode, and pose 1 was selected for each system. In these systems, the most plausible pose was selected based on internal consistency across the top poses and predicted binding energy. Minor differences in initial docking orientation are expected to be fine-tuned during the subsequent 500 ns simulations.

### Conformational Stability and Fluctuations

**Fig. 2:**
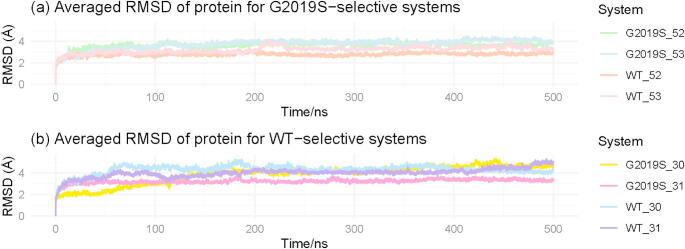
The averaged root-mean standard deviation (RMSD) of three parallel trajectories for **a** protein in G2019S-selective systems and **b** protein in wild-type-selective systems

The ligand-kinase complexes exhibited varying degrees of stability, as shown in Figs. 2, S1 and S2. G2019S-selective systems effectively achieved stabilisation in the final 100 ns of the simulation (Fig. 2a). In particular, the G2019S-selective compounds 53 and 52 in the G2019S kinase exhibit larger protein RMSD than the wild-type, indicating that the G2019S kinases undergo more conformational changes than the wild-type when binding to the G2019S-selective inhibitors. Similarly, wild-type-selective systems also obtained the stabilisation, particularly during the last 100 ns, as shown in Fig. 2b. The compound 31, which showed selectivity towards the wild-type, demonstrated a higher protein RMSD in the wild-type, similar to compounds 53 and 52, which are selective for G2019S, induced more conformational fluctuations instead of stabilising the complexes. On the contrary, compound 30 showed less distinction between the variants in the protein RMSD.

To determine potential binding patterns associated with the lowest free energy of binding, the RMSDs of the ligands, representing their conformational changes, and the distance between the mass centres of the ligand and Gly/Ser 2019 (the mutation site) were analysed and converted into free energy landscapes (FELs). The FELs (Fig S3) illustrate the converged tendency to the only stable funnel area in all eight systems, reflecting that these ligand-kinase complexes hold only one thermodynamically and kinetically stable binding state.

As shown in Fig S4, the essential dynamic motions captured by principal components analysis (PCA) illustrate that all systems converged during the simulation. The first two principal components in all eight systems account for over 50% of the total observed movements, indicating that the systems are not undergoing significant, unpredictable changes. The simulation frames are colour-coded, transitioning from red at the beginning to purple at the end. As the simulations progress, the systems gradually converge into smaller regions of conformational space, reflecting a transition from unstable to stable equilibrium states, as projected onto the first two dominant principal components.

### Selective Inhibitors Disrupt the Structural Integrity of Their Specific Kinases

**Fig. 3:**
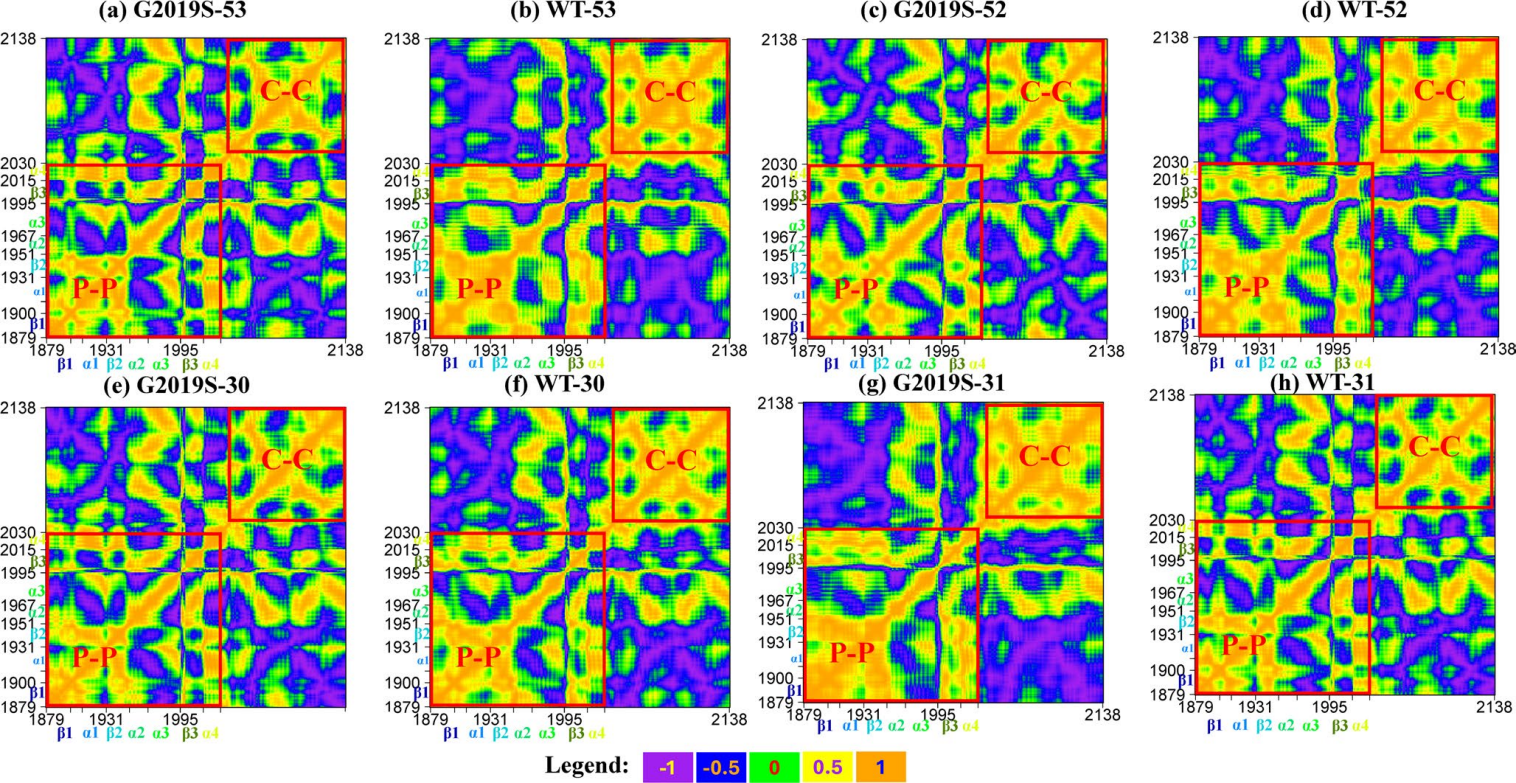
Dynamic cross-correlation matrices (DCCM) for the crucial interval of **a** compound 53 in G2019S kinase; **b** compound 53 in wild-type kinase; **c** compound 52 in G2019S kinase; **d** compound 52 in wild-type kinase; **e** compound 30 in G2019S kinase; **f** compound 30 in wild-type kinase; **g** compound 31 in G2019S kinase; **h** compound 31 in wild-type kinase. Positive, non-relative, and negative values are represented by orange, yellow, green, blue, and purple, respectively. P–P: pocket–pocket; C–C: C-terminal–C-terminal

During the simulation, the ligand and the kinase complemented each other through various conformational changes. To visualise the impact of different ligands on the pocket and overall conformations, the dynamic cross-correlation matrices (DCCM) across all systems were presented in Fig. 3a- h. In these DCCMs, residue pairs that move in the same direction are indicated by positive values (orange), while those that move in opposite directions, displaying anti-correlated motion, are indicated by negative values (purple). Residue pairs with no statistical correlation are represented by values close to zero. Strong positive correlations indicate cooperative residue movement trends, while negative correlations between regions may reflect damage to the structural integrity and stability in different moving orientations [45].

In the DCCM (Fig. 3a -d) of G2019S-selective systems (compounds 53 and 52), the internal correlations within the pocket regions and nearby residues (P-P) and within the C-terminal (C-C) regions in G2019S are less than those of the wild-type. This indicates that the atoms or residues in these regions demonstrate inconsistent cooperative motion during the simulation. Conversely, the wild-type selective compound 31 reduces positive internal correlations within the P-P and C-C regions in the wild-type kinase (Fig. 3g and h). However, as a weaker selective inhibitor, the effects observed with compound 30 are less pronounced (Fig. 3e and f).

In summary, selective inhibitors disrupt the integrity of their specific kinases. These conformational changes reduce the integrity of the protein, probably leading to its inactivation or degradation.

### Selective Inhibitors Break the Asp2017-Glu1920 Interaction

**Fig. 4:**
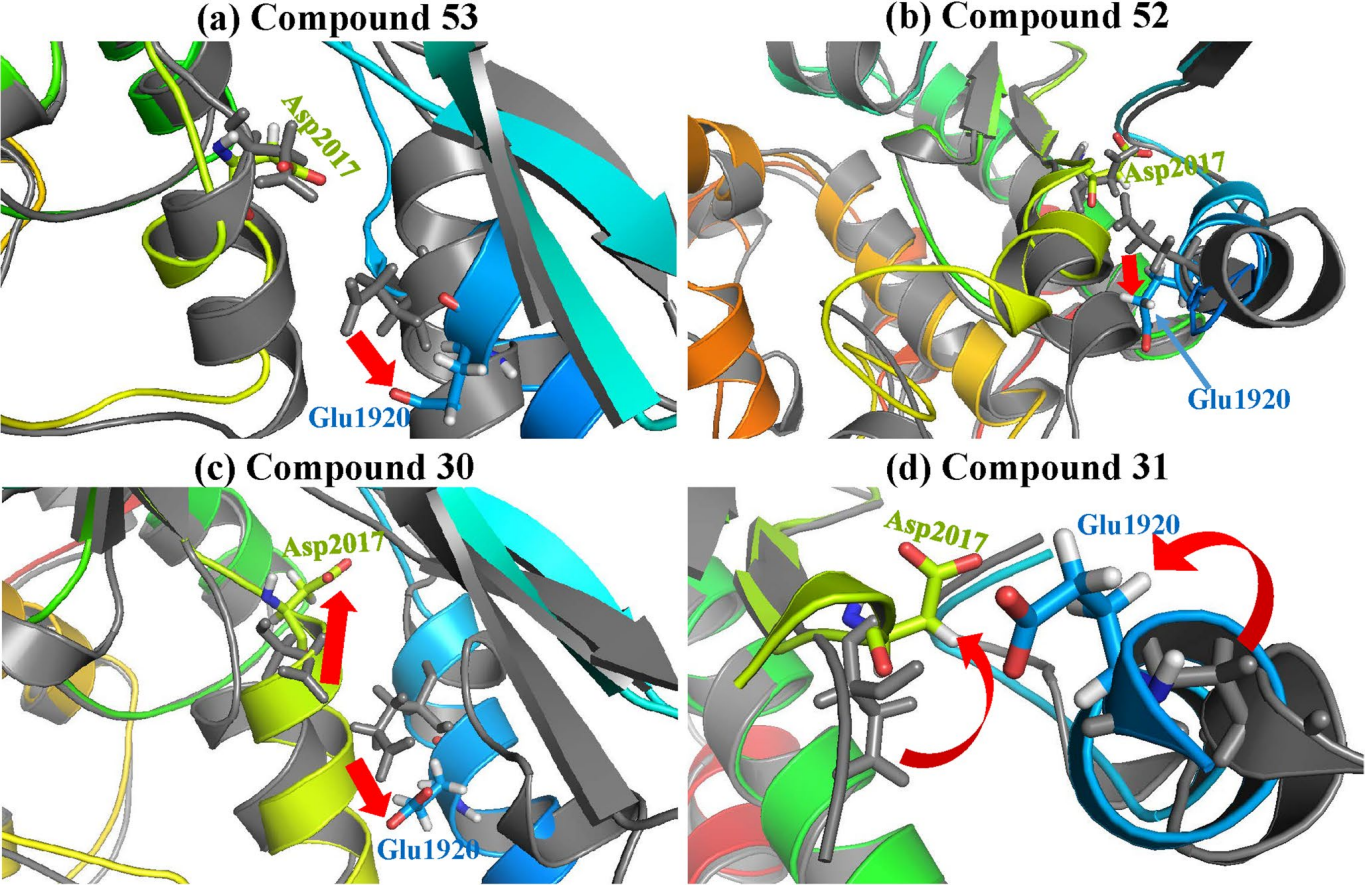
Conformations of Asp2017-Glu1920 in representative conformations for **a** compound 53, **b** compound 52, **c** compound 30, and **d** compound 31. The G2019S-ligand complex was aligned with the wild-type-ligand complex, and the G2019S was painted colourful, and the wild-type was grey

Asp2017 and Glu1920, positioned at the α4 and α1 helices, contribute to the binding of Mg^2+^ and the γ-phosphate of ATP, where the catalytic process occurs [14]. The separation of Asp2017 and Glu1920 may impair the kinase’s phosphorylation activity. For G2019S-selective inhibitors, the conformation of Asp2017 in G2019S and wild-type overlapped, but Glu1920 rotated away in G2019S (Fig. 4a and b). Compound 31 induced rotations that obstruct Asp2017 and Glu1920 from making contact in the wild-type (Fig. 4d). However, the less-selective compound 30, which was expected to favour wild-type kinase, showed the opposite effect, bringing the wild-type Asp2017 and Glu1920 closer than in G2019S (Fig. 4c).

The selective inhibitors cause an increasing distance between the mass centres of Asp2017 and Glu1920 across the last 100 ns of the simulations. Specifically, G2019S-selective inhibitors (compounds 53 and 52) in the G2019S system result in a greater separation of Asp2017 and Glu1920 than in the wild-type system (Fig S5a). Similarly, the wild-type-selective compound 31 causes a greater separation of Asp2017 and Glu1920 in the wild-type system than in the G2019S system (Fig S5b). Conversely, compound 30 did not exhibit this effect (Fig S5b), indicating its inability to inactivate the kinase by disrupting the Asp2017-Glu1920 interaction, which corresponds to its conformation in Fig. 4c.

### Binding Patterns of Selective Inhibitors

**Fig. 5:**
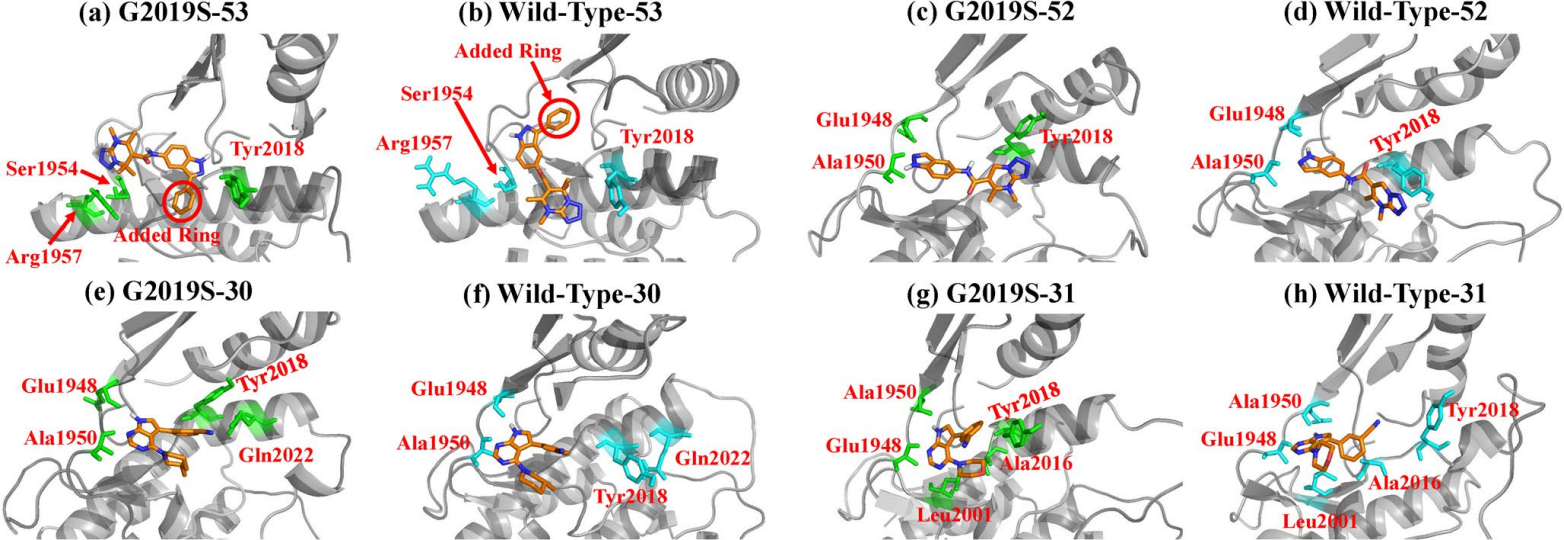
Binding conformations of the representative frames in G2019S-selective systems: **a** compound 53 in G2019S kinase; **b** compound 53 in wild-type kinase; **c** compound 52 in G2019S kinase; **d** compound 52 in wild-type kinase; **e** compound 30 in G2019S kinase; **f** compound 30 in wild-type kinase; **g** compound 31 in G2019S kinase; **h** compound 31 in wild-type kinase

The predicted binding modes of G2019S-selective inhibitors (compounds 52 and 53) show noticeable differences when bound to the G2019S mutant versus the wild-type LRRK2 kinase, suggesting that the single G2019S mutation may influence ligand orientation or interactions within the binding pocket. Compound 53, featuring a dihydrotetrazolo[1,5-a] pyrimidine scaffold, is more selective to G2019S than compound 52. As illustrated in Fig. 5a and b, compound 53 binds to the G2019S and wild-type kinase in markedly different orientations. This variation primarily arises from the π-π stacking interactions between the dihydrotetrazolo[1,5-a]pyrimidine ring and Arg1957 in G2019S, which are absent in the wild-type. Additionally, compound 53 forms a hydrogen bond with Ser1954 in G2019S, an interaction not observed in the wild-type. In the wild-type, the compound established a hydrogen bond with His1998, whereas in G2019S, His1998 is involved solely in a van der Waals interaction (Fig S6). Furthermore, in G2019S, the 2-azole ring forms a hydrogen bond with Tyr2018, an interaction not found in the wild-type. However, Tyr2018 still contributes positively to the binding free energy in the wild-type, with a value of -0.99 kcal/mol (Table S3).

Despite the significant binding pattern differences of compound 53 in G2019S and wild-type, the interacting residues within the binding pocket remain primarily unchanged (Fig S6), such as Ser1889, Leu2001, and Ala2016. This may also contribute to the similar general pattern observed in the DCCMs (Fig. 3a and b) with only minor differences in the intensity. The selectivity of compound 53 for the G2019S is attributed to the variation of interaction types with the key residues. For instance, Tyr2018 forms hydrogen bonds with ligands in the G2019S. In contrast, the ligand adopts an opposite orientation in the wild-type kinase and remains further from Tyr2018, resulting in weaker, non-specific van der Waals interactions.

Compound 52 did not exhibit any major differences in its binding orientations between the G2019S and the wild-type kinase, as shown in Fig. 5c and d. In both kinases, the double-bonded nitrogen in the 2-azole ring acts as a hydrogen bond acceptor from the imino group of Ala1950. However, the differing orientations of the 2-azole ring led to distinct interactions in the wild-type kinase. The ring’s conformation permits a hydrogen bond donation to the carbonyl of Glu1948; in the G2019S kinase, the polar hydrogen rotates away and is no longer positioned to interact with Glu1948. Additionally, a slight rotation of the dihydrotetrazolo[1,5-a]pyrimidine ring in the wild-type enables π-π stacking with Tyr2018, but the G2019S Tyr2018 shifts upward and moves further away from the ligand.

The scaffolds of compounds 52 and 53 are highly symmetric along the left-right axis, which likely explains why the docking results produced multiple poses with opposing orientations. In compound 53, the addition of an aromatic ring to the indazole moiety breaks this symmetry and contributes to its G2019S-selectivity. Compound 52, lacking the additional aromatic ring, fits well into both the wild-type and G2019S kinases with similar binding patterns. However, the extended structure of compound 53 prevents it from fitting into the pocket as compound 52 does, prompting it to explore alternative binding modes. In the wild-type kinase, the ligand tilts slightly upward to accommodate the added ring, while in G2019S, it adopts an entirely different orientation. This orientation shift in G2019S does not significantly disrupt binding, likely due to the inherent symmetry of the scaffold, and instead allows the ligand to access a nearby cavity to accommodate the added ring. Interestingly, Compound 53 cannot adopt the same orientation in the wild-type kinase as it does in G2019S, likely due to steric hindrance from residues in the β1 strand (Fig S7).

The overall binding orientations are largely conserved for the wild-type selective inhibitors, with only subtle variations responsible for driving selectivity. Based on the IC_50_ values, compound 31 demonstrates higher activity and selectivity than compound 30 towards wild-type. Compound 30 adopts a similar binding conformation in both G2019S and wild-type kinases, as shown in Fig. 5e and f. It forms two hydrogen bonds with the β2 in the G2019S and wild-type: one between the pyrrolic nitrogen and Glu1948 and another between the pyridine nitrogen and Ala1950. In the G2019S, the α4 interacts with the ligand via the cyano group, forming a hydrogen bond with Gln2022, while Tyr2018 engages in van der Waals interactions (Fig S6). However, in the wild-type kinase, the downward shift of Tyr2018 blocks the potential interaction between Gln2022 and the ligand.

For compound 31, the ether substitution alters its binding pattern (Fig. 5g and h). The oxygen atom donates electrons, increasing the electron density across the entire conjugated system. As a result, the nitrogen-driven hydrogen bonds with Ala1950 and Glu1948 are strengthened, contributing more to the binding free energy compared to compound 30, as shown in Table S3 (highlighted in cyan). In the wild-type kinase, Leu2001 and Ala2016 participate in π-π stacking interactions, whereas these interactions are dismissed in G2019S due to the increased distance between the residues. Additionally, Tyr2018 rotated away from Ala2016, remaining involved in a weak van der Waals interaction in G2019S but no longer engaged in the wild-type kinase (Fig. 5g-h, Fig S6, and Table S3).

### Insight from per-residue Energy Decomposition

**Fig. 6:**
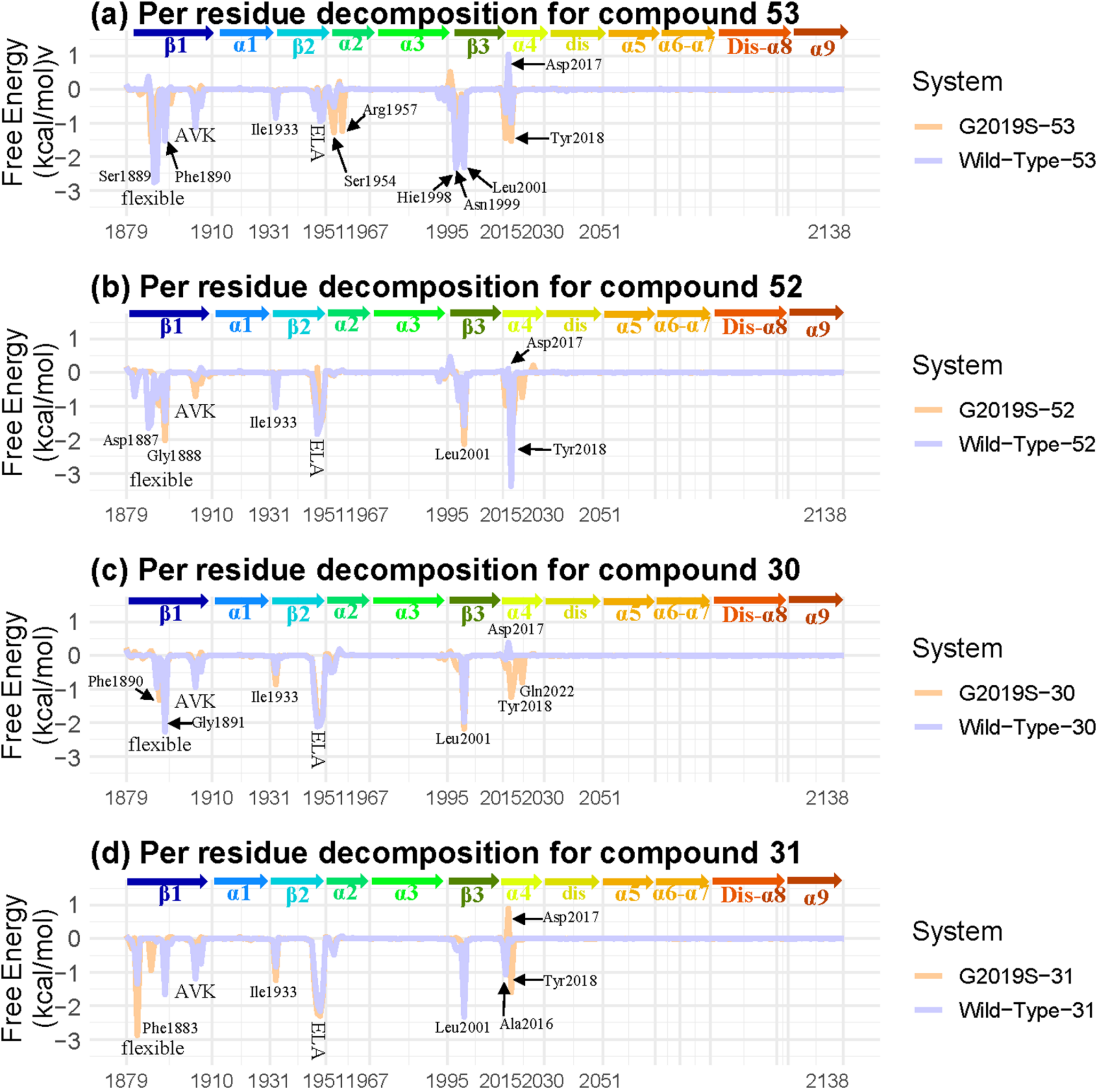
Per residue energy decomposition of the MM/GBSA calculation in the crucial interval for **a** Compound 53, **b** Compound 52, **c** Compound 30, and **d** Compound 31. ELA: Glu1948, Leu1949, and Ala1950. AVK: Ala1904, Val1905, and Lys1906. The free energy of significant residues is listed in Table S2

The per-residue energy decomposition (Fig. 6) illustrates the contributions of individual residues to the ligand-kinase binding. The overall shape of the decomposition shows a similar trend across all systems, highlighting the resemblance between their binding sites. As this study focuses solely on the kinase domain of LRRK2, excluding all other domains, the initial portion of the β1 segment in the N-terminal, which is typically connected to the COR domain of LRRK2, was left unconstrained. This led to significant regional flexibility, resulting in considerable variability in binding free energy (Fig. 6) and RMSF (Figs S1 and S2).

In all systems, the large side chain of Ile1933, located in the disordered region between α1 and β2, contributes significantly to binding, more so than the surrounding residues. The N-terminal segment of β2 plays a crucial role in forming the binding pocket, where the ELA region (Glu1948, Leu1949, and Ala1950) consistently shows high free energy contributions across all eight systems. Leu2001, a key component of the catalytic spine [14], was also involved in binding in every system. The G-loop at the C-terminal of β1 also played a significant role, with the AVK region (Ala1904, Val1905, and Lys1906) contributing significantly to all systems.

The per-residue contribution analysis in DYG/S reveals a distinct pattern, providing valuable insights into the selective properties of binding. In the case of G2019S-selective molecules (compounds 53 and 52), Tyr2018 contributes to binding in G2019S and wild-type kinases (Fig. 6a and b). However, for compounds 30 and 31, the interaction with Tyr2018 in the wild-type is negligible, while in G2019S, it is maintained through weak van der Waals forces (Fig S6). These differences, although subtle, may serve as a criterion for identifying potential G2019S-selective inhibitors. Asp2017 favours the binding of G2019S-selective compounds 53 and 52 on the G2019S, while their binding to the wild-type is unfavourable (Table S3, highlighted in red and yellow). For compound 31, which exhibits strong wild-type selectivity based on IC_50_, the free energy decomposition for Asp2017 is negative in the wild-type kinase and positive in the G2019S kinase (Table S3). However, this phenomenon was absent in the case of compound 30 (**Table S3**).

In conclusion, the per-residue decomposition reveals several key residues involved in binding. Across all systems, Ile1993, Leu2001, the ELA residues (Glu1948, Leu1949, and Ala1950), and the AVK residues (Ala1904, Val1905, and Lys1906) consistently contributed favourably to binding. G2019S-selective inhibitors strongly prefer Tyr2018, whereas wild-type-selective inhibitors engage Tyr2018 only in the G2019S kinase. Moreover, the role of Asp2017 aligns with the selectivity of these inhibitors, as it influences binding preferences between G2019S and wild-type kinases.

### Effects of the Gly/Ser Mutation on the Ligand Selectivity

The compound 53 achieves selectivity via distinct binding orientations, while compound 30 shows limited selectivity. To isolate the effect of the Gly-Ser mutation, we analysed compounds 52 and 31, where ligands bind in similar orientations, to assess how the mutation alters nearby residues.

**Fig. 7:**
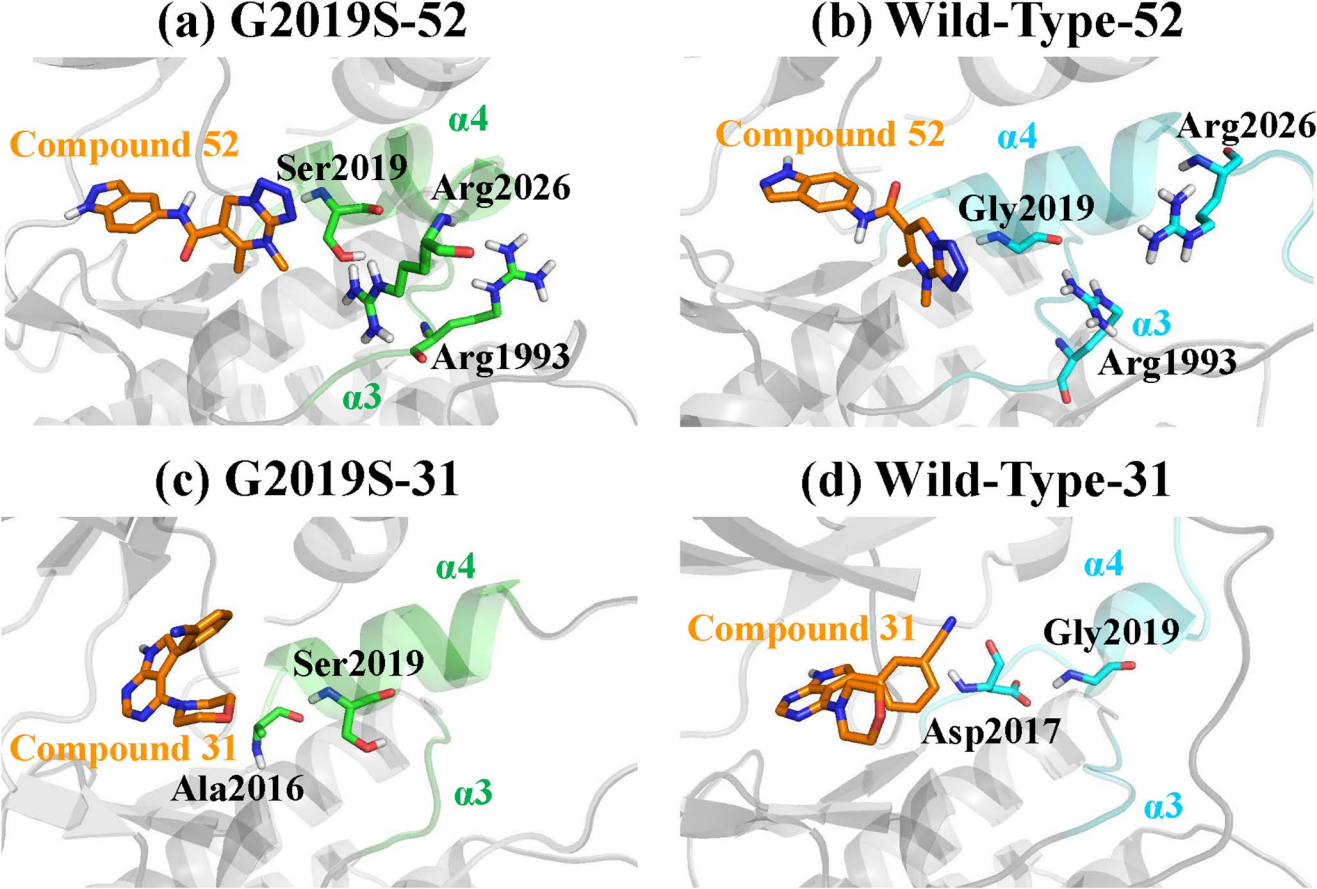
Structural consequences of the Gly-2019-Ser mutation in **a**, **b** compound 52 and **c**, **d** compound 31 systems

A previous study reported that hydrogen bonds between Ser2019 and residues in α3, such as Lys1996, play a stabilising role in maintaining the active conformation of the ATP-binding pocket [46]. Building on this observation, as shown in Fig. 7a–b and Fig S8a–b, in the wild-type-compound 52 system, Gly2019 in the α4 helix forms a hydrogen bond with Arg1993 in α3, stabilising the α3–α4 interface. Upon mutation to Ser2019, the introduced hydroxyl oxygen instead forms a hydrogen bond with Arg2026 within the same helix, thereby disrupting the original contact with α3. This shift promotes separation between α3 and α4, destabilising the kinase pocket architecture. In the context of compound 52, this structural rearrangement favours its binding to the G2019S mutant over the wild-type kinase, contributing to its observed selectivity.

In the compound 31 systems, the Gly-to-Ser mutation does not enable the introduced hydroxyl group of Ser2019 to form any strong interactions with α3; instead, only weak van der Waals contacts with several α3 residues are observed (Fig. S8c–d). However, these van der Waals forces appear to pull Ser2019 downward, leading to a shift in hydrogen bonding acceptor from Asp2017 in the wild-type to Ala2016 in the G2019S (Fig. 7c–d). Combining this with the per-residue binding free energy analysis in Fig. 6d, it becomes evident that Ala2016 contributes favourably to ligand binding in the wild-type system. In G2019S, the introduced Ser2019 occupies the position of Ala2016, which normally contributes favourably to ligand binding, and releases Asp2017, whose contribution is unfavourable. Thereby, the Gly-Ser weakens the overall binding affinity to the compound 31.

The introduction of a polar hydroxyl group by the Gly-to-Ser mutation disrupts the intrinsic flexibility of the DYG ψ motif, leading to conformational rearrangements near residue 2019 [47]. These local structural changes contribute to the differential selectivity observed for compounds 52 and 31. When binding to compound 52, Gly to Ser substitution influences the interaction between the α3–α4 contacts. In contrast, in compound 31 complexes, Gly2019 and Ser2019 could occupy by interacting with Asp2017 and Ala2016, respectively, potentially altering local residue contacts and contributing differently to ligand binding. These distinct effects may help explain the observed selectivity.

### Selectivity Mechanism Beyond Conventional Competitive Inhibition

Conventional competitive inhibition occurs when an inhibitor competes with ATP for the active site [17, 48]. Selectivity in competitive inhibitors typically relies on differences in binding pockets. The structural similarity between G2019S and wild-type kinases presents a significant challenge in designing mutation-selective inhibitors [4]. Despite this, several works have reported IC_50_ values that indicate selectivity for the G2019S or wild-type [9], with the mechanism requiring further investigation.

Interestingly, the binding free energy (Table 1) reveals an inverse trend in selectivity, where the selective inhibitors exhibit poorer binding with their specific kinases. Compounds 53 and 52, expected to bind to the G2019S kinases strongly, show unexpectedly inconsistent results that bind tightly to the wild-type than to G2019S. Compound 30 shows more negative binding free energy with G2019S kinases than the wild-type, while compound 31 exhibits comparable free energy in the wild-type (-33.51 kcal/mol) and G2019S (-32.37 kcal/mol). Binding free energy results contradict experimental selectivity, with stronger predicted affinities for wild-type despite G2019S-preferred IC₅₀ values. These inconsistencies highlight the complexity of LRRK2-inhibitor interactions and suggest that factors beyond static binding energy, such as conformational dynamics or allosteric effects, may play a pivotal role in selectivity.

**Table 1:** Predicted binding free energies for crucial intervals (100 frames) in all systems (kcal/mol)

Systems	∆E_vDW_	∆E_eel_	∆E_GB_	∆E_Surf_	∆G_gas_	∆G_solv_	∆G_binding_
GS-53	− 47.91 ± 2.95	− 36.47 ± 5.08	50.08 ± 3.42	− 4.22 ± 0.16	− 84.38 ± 5.42	45.86 ± 3.37	− 38.52 ± 3.11
WT-53	− 54.29 ± 2.77	− 38.30 ± 4.43	49.66 ± 3.49	− 4.33 ± 0.11	− 92.59 ± 4.73	45.33 ± 3.46	− 47.26 ± 2.92
GS-52	− 43.23 ± 2.41	− 21.84 ± 3.77	39.75 ± 3.05	− 3.66 ± 0.11	− 65.07 ± 4.53	36.09 ± 3.01	− 28.97 ± 2.61
WT-52	− 44.42 ± 2.24	− 40.14 ± 5.20	52.63 ± 4.23	− 3.59 ± 0.10	− 84.56 ± 5.41	49.04 ± 4.19	− 35.52 ± 2.54
GS-30	− 44.87 ± 2.30	− 28.63 ± 4.31	38.87 ± 3.80	− 3.72 ± 0.06	− 73.50 ± 4.26	35.15 ± 3.80	− 38.36 ± 2.57
WT-30	− 35.05 ± 2.57	− 21.31 ± 3.76	29.45 ± 3.35	− 2.87 ± 0.13	− 56.36 ± 3.20	26.58 ± 3.36	− 29.78 ± 2.17
GS-31	− 35.17 ± 2.40	− 24.76 ± 3.73	30.68 ± 3.33	− 3.12 ± 0.11	− 59.93 ± 3.63	27.56 ± 3.32	− 32.37 ± 2.48
WT-31	− 35.29 ± 2.45	− 27.64 ± 3.08	32.55 ± 2.32	− 3.12 ± 0.13	− 62.94 ± 3.28	29.43 ± 2.31	− 33.51 ± 2.51

∆E_vdW_: van der Waals energy; ∆E_ele_: Electrostatic energy; ∆G_GB_: Solvation-free energy change part based on the Generalised Born model; ∆E_Surf_: Free energy change contributed by the surface area; ∆G_gas_: Gas phase free energy change; ∆G_solv_: Solvation free energy change; ∆G_binding_: Total free energy change

The mechanism of selective inhibition is likely not driven by directly binding or stabilising a specific target, albeit these inhibitors occupy the ATP site. Among the compounds examined, compound 31 was the only one to exhibit a binding affinity trend consistent with its experimental selectivity, showing stronger binding to the wild-type (-33.51 kcal/mol) kinase than to the G2019S (-32.37 kcal/mol) slightly, which was driven by local conformational changes around the DYG motif, alternating interactions with Ala2016/Asp2017 as discussed in Sect. 3.7. Although these ligands remain tightly bound to the ATP pocket, they do not achieve selectivity between wild-type and G2019S based on binding affinity. Instead, their selectivity stems from their ability to induce conformational shifts that alter the kinase’s active state dynamics. By modulating the conformational landscape of the kinases, as discussed in Sect. 3.3 and 3.4, these inhibitors promote an inactive or less active conformation, achieving inhibition through dynamic regulation rather than relying solely on binding strength.

Similar mechanisms have already been reported and defined as “inhibitor-induced kinase degradation” for situations where inhibitors might destabilise kinases [49]. For example, 5Z7O binds to the ATP site of MAP2K but inhibits it by regulating conformational changes in the ATP pocket, rather than through conventional competitive inhibition [50]. In another report, Vertex-11e could remain at the ATP site in ERK1/2, inducing a conformational state of the kinase that leads to inactivation [51]. The LRRK2 selective inhibitors in this study are more characteristic of inhibitor-induced kinase degradation/destabilisation than conventional competitive inhibition [52]. This observation warrants further investigation.

## Conclusion

LRRK2 kinase conformational plasticity has been extensively studied, with significant insights captured when type I or II inhibitors bind to it [47, 53, 54]. The kinase domain not only functions as a dynamic allosteric hub for LRRK2 activation [55], but also undergoes inhibitor-induced conformational changes critical for drug discovery [14, 15]. This principle can be applied to designing selective inhibitors for G2019S vs. wild-type LRRK2, where distinct conformational preferences provide insight into the structural features necessary for selective inhibition.

This study employed molecular dynamics simulations to investigate the potential mechanisms underlying the selectivity of LRRK2 inhibitors towards G2019S or wild-type kinases. The simulations revealed that selectivity is not achieved through more favourable binding affinities. Instead, conformational changes in the kinase, such as reduced protein integrity and the separation between Asp2017 and Glu1920, likely lead to kinase inactivation. Specifically, compound 53 binds to G2019S in a unique orientation compared to the wild-type. Additionally, compounds 52 and 31 also show distinct binding patterns, though they maintain a similar overall orientation. Additionally, key residues, such as Ala1950, contribute significantly to binding in all systems, while Tyr2018 and Asp2017 are likely drivers of selectivity, from which future designs of LRRK2 mutant-selective inhibitors might benefit.

However, this study still has several limitations. It focused exclusively on the kinase domain, excluding other domains such as COR and ROC, which might have influenced kinase behaviour such as β1 flexibility. Besides, the limited number of inhibitors and mutations analysed may not fully capture the complete range of selective mechanisms. Furthermore, compound 30 exhibited less reliable binding modes from modelling and minimal selectivity from simulation, requiring further investigation into its binding mechanism.

While designing selective inhibitors for proteins with similar active sites is challenging, the inhibitor-specific conformational dynamics demonstrated in this work illustrate that successful drug design requires a thorough understanding of protein dynamics. When ligand selectivity is driven by conformational changes rather than direct competitive binding, it presents both challenges and opportunities for drug development, particularly in the discovery of selective LRRK2 inhibitors [56].

Our findings underscore the critical importance of accounting for protein conformational dynamics when interpreting ligand binding modes, particularly in cases where inhibition results from a ligand’s ability to induce or stabilise distinct conformational states, rather than from conventional competitive mechanisms. This challenges the classical “lock-and-key” paradigm and reinforces the relevance of dynamic binding models, such as “induced fit” and “conformational selection,” in rational drug design. Incorporating these principles offers a more physiologically realistic framework for the development of effective therapeutics.

## Supplementary Information

Below is the link to the electronic supplementary material.

Supplementary Material 1

## Data Availability

The datasets generated during and/or analysed during the current study are available upon request to the corresponding author.

## Declarations

### Type

Original Article.
