# Supplementary material for: Comparative Molecular Dynamics Reveals How LRRK2 Inhibitors Distinguish G2019S from Wild-Type
Source: Neurochem Res. 2025 Aug 13;50(4):259. doi: 10.1007/s11064-025-04520-w (PMC12350454; doi:10.1007/s11064-025-04520-w)
Supplement: Supplementary file 1 — Supplementary Material 1 [file 11064_2025_4520_MOESM1_ESM.docx]

**Comparative Molecular Dynamics Reveals How LRRK2 Inhibitors Distinguish G2019S from Wild-Type**

| **Table S1** Top 5 predicted binding modes from molecular docking foe vey system. | | | | | | | | | | | |
| --- | --- | --- | --- | --- | --- | --- | --- | --- | --- | --- | --- |
| **GS**  **System** | **No** | Affinity  (kcal/mol) | RMSD l.b. | RMSD u.b. | Ligand Conformation | **WT**  **System** | **No** | Affinity  (kcal/mol) | RMSD l.b. | RMSD u.b. | Ligand Conformation |
| **53** | **1** | -9.2 | 0.000 | 0.000 | 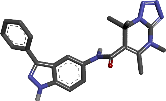 | **53** | **1** | -8.9 | 0.000 | 0.000 | 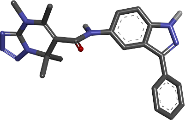 |
|  | **2** | -8.8 | 3.409 | 6.023 | 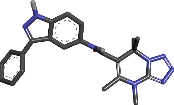 |  | **2** | -8.7 | 5.196 | 7.744 | 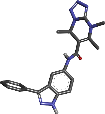 |
|  | **3** | -8.8 | 3.371 | 6.344 | 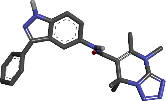 |  | **3** | -8.6 | 2.592 | 4.438 | 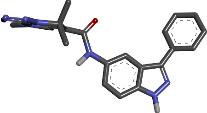 |
|  | **4** | -8.8 | 3.732 | 6.338 | 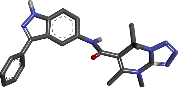 |  | **4** | -8.6 | 2.513 | 4.157 | 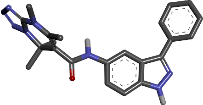 |
|  | **5** | -8.7 | 2.849 | 8.434 | 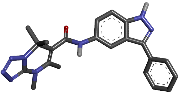 |  | **5** | -8.6 | 2.179 | 8.777 | 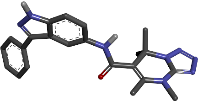 |
| **52** | **1** | -8.4 | 0.000 | 0.000 | 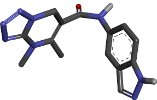 | **52** | **1** | -8.3 | 0.000 | 0.000 | 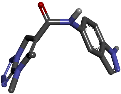 |
|  | **2**  ***** | -8.1 | 12.356 | 14.950 | 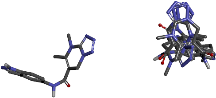 |  | **2** | -8.2 | 2.953 | 4.136 | 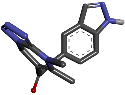 |
|  | **3** | -8.0 | 2.025 | 2.939 | 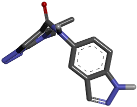 |  | **3** | -8.1 | 2.284 | 3.359 | 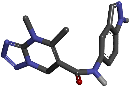 |
|  | **4** | -8.0 | 2.070 | 3.014 | 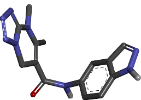 |  | **4** | -8.0 | 0.571 | 1.358 | 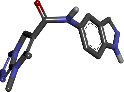 |
|  | **5** | -8.0 | 1.215 | 2.027 | 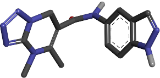 |  | **5** | -7.7 | 0.865 | 0.909 | 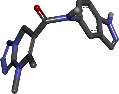 |
| **30** | **1** | -8.1 | 0.000 | 0.000 | 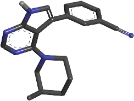 | **30** | **1**  **3**  **5**  ***** | -8.2  -8.0  -7.5 | 0.000  0.770  2.104 | 0.000  0.882  5.393 | 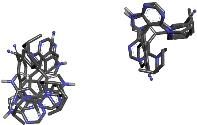 |
|  | **2** | -8.0 | 2.162 | 4.474 | 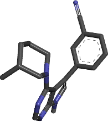 |  | **2** | -8.0 | 12.979 | 15.795 | 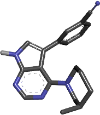 |
|  | **3** | -8.0 | 1.769 | 4.098 | 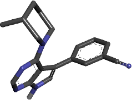 |  | **4** | -7.7 | 12.417 | 14.117 | 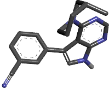 |
|  | **4** | -7.6 | 2.683 | 6.163 | 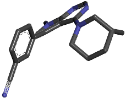 |  | **6** | -7.4 | 12.722 | 15.880 | 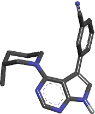 |
|  | **5**  ***** | -7.6 | 15.759 | 18.959 | 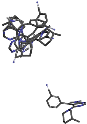 |  | **7** | -7.3 | 11.897 | 14.720 | 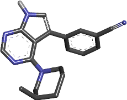 |
| **31** | **1** | -7.8 | 0.000 | 0.000 | 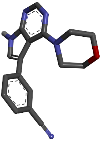 | **31** | **1** | -7.8 | 0.000 | 0.000 | 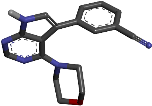 |
|  | **2** | -7.8 | 2.202 | 3.893 | 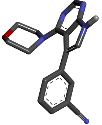 |  | 2 | -7.6 | 2.333 | 2.748 | 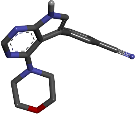 |
|  | **3** | -7.6 | 2.757 | 5.957 | 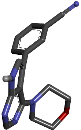 |  | 3 | -7.5 | 2.808 | 5.816 | 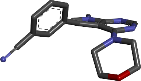 |
|  | **4** | -7.6 | 2.167 | 3.449 | 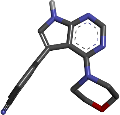 |  | 4 | -7.4 | 1.566 | 1.750 | 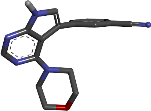 |
|  | **5** | -7.6 | 2.427 | 3.812 | 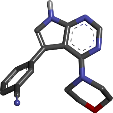 |  | 5 | -7.3 | 2.201 | 3.925 | 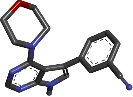 |

Note: * Some of these poses were located outside the binding pocket, as illustrated in the figures showing both inside and outside poses.


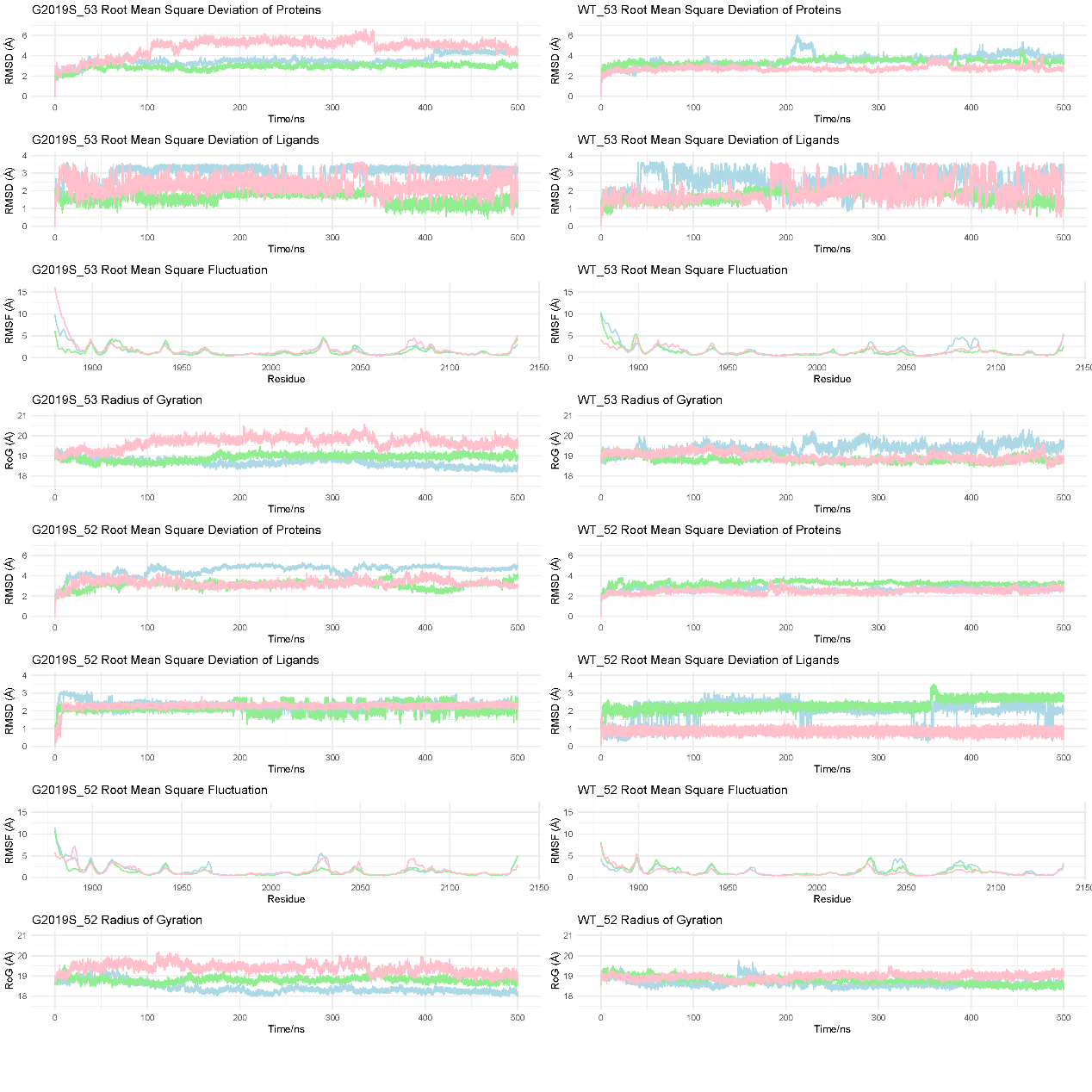


**Fig. S1** Trajectories analysis (RMSD, RMSF, and RoG) for compounds 53 and 52 in complex with G2019S/wild-type LRRK2 kinase.


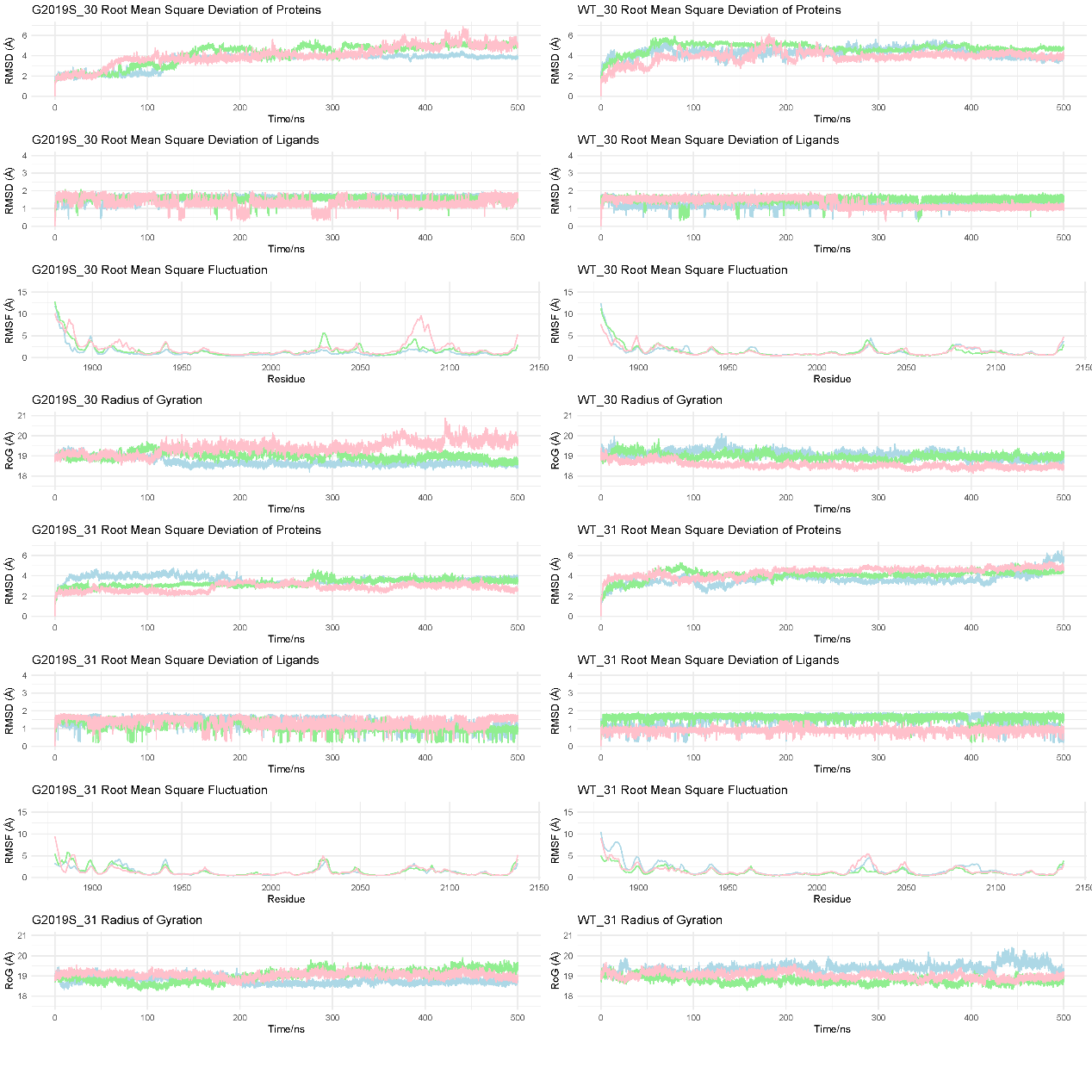


**Fig. S2** Trajectories analysis (RMSD, RMSF, and RoG) for compounds 30 and 31 in complex with G2019S/wild-type LRRK2 kinase.

**Table S2** Details about the ground minima in all systems.

| Target | Ligand | Range of RMSD /Å | Range of Distance /Å | Crucial Interval /ns | Centre /ns |
| --- | --- | --- | --- | --- | --- |
| GS | 53 | [1.179, 1.232] | [12.52, 12.68] | [483.97, 484.96] | 484.39 |
| WT | 53 | [1.473, 1.528] | [11.24, 11.38] | [415.71, 416.70] | 416.26 |
| GS | 52 | [2.223, 2.261] | [7.852, 8.004] | [465.59, 466.58] | 466.07 |
| WT | 52 | [2.724, 2.750] | [9.376, 9.493] | [475.01, 476.00] | 475.48 |
| GS | 30 | [1.632, 1.653] | [8.053, 8.191] | [450.44, 451.43] | 450.93 |
| WT | 30 | [1.577, 1.622] | [14.70, 14.86] | [416.38, 417.37] | 416.89 |
| GS | 31 | [1.232, 1.286] | [10.06, 10.22] | [423.86, 424.85] | 424.58 |
| WT | 31 | [0.892, 0.924] | [14.81, 14.91] | [438.00, 438.99] | 438.50 |

Note: GS: G2019S; WT: wild-type. The crucial interval reflects to the simulation duration of 1 ns that contains most of the frames in the minima; the centre frame reflects to a specific frame that could represent the overall conformation of the interval.


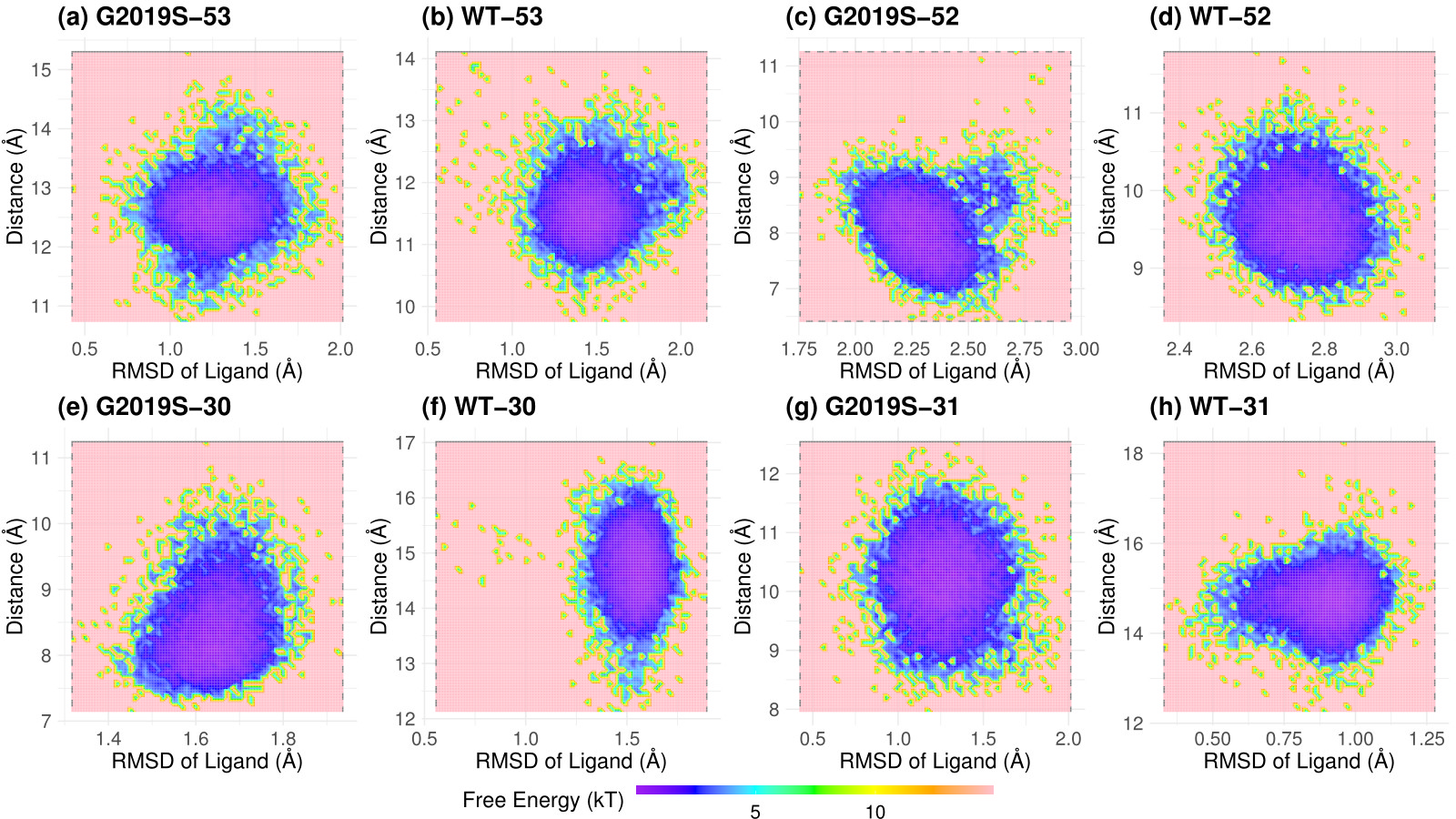


**Fig. S3** Free energy landscape (FEL), which was converted from (1) the RMSDs of the ligands and (2) the distances between the mass centres of Gly/Ser 2019 and the ligands for all systems. The unit for the free energy in the landscapes is kT, k (Boltzmann constant) = 1.38× 10 ^-23^ J/K, T (temperature) stabilised around 310 K during the simulations.


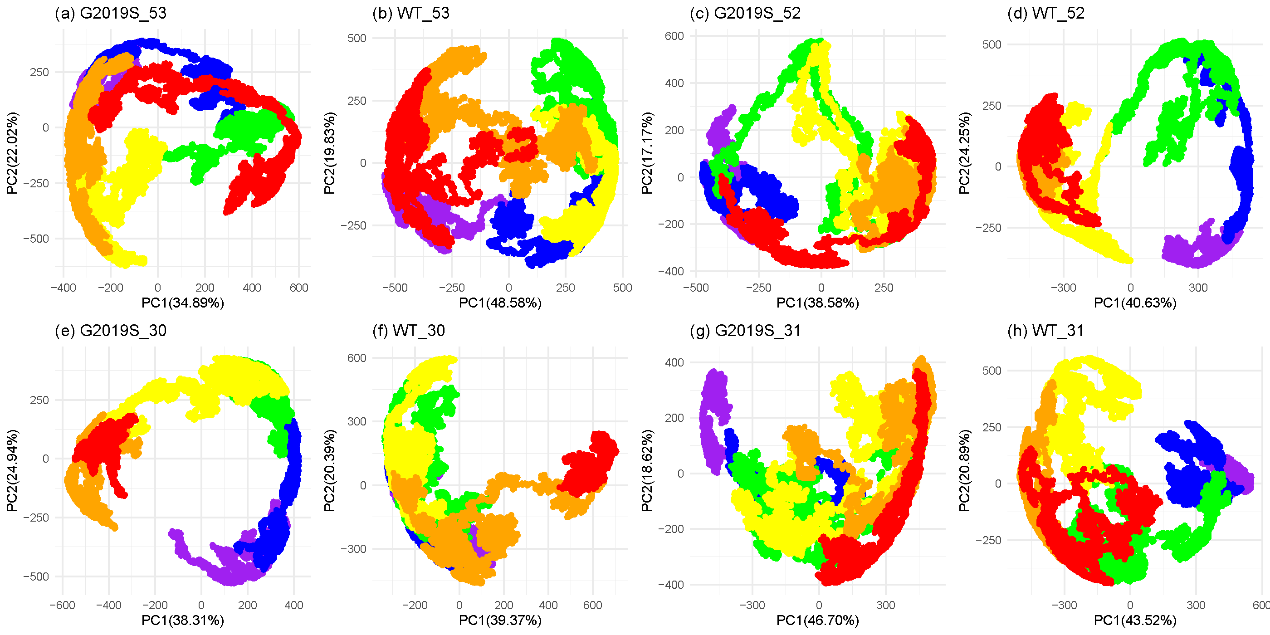


**Fig. S4** (a-h) Projections of the first two principal components (PC1 and PC2) and the percentage of the motion. The colour gradient in red, orange, yellow, green, and purple along the progression of the simulation time.


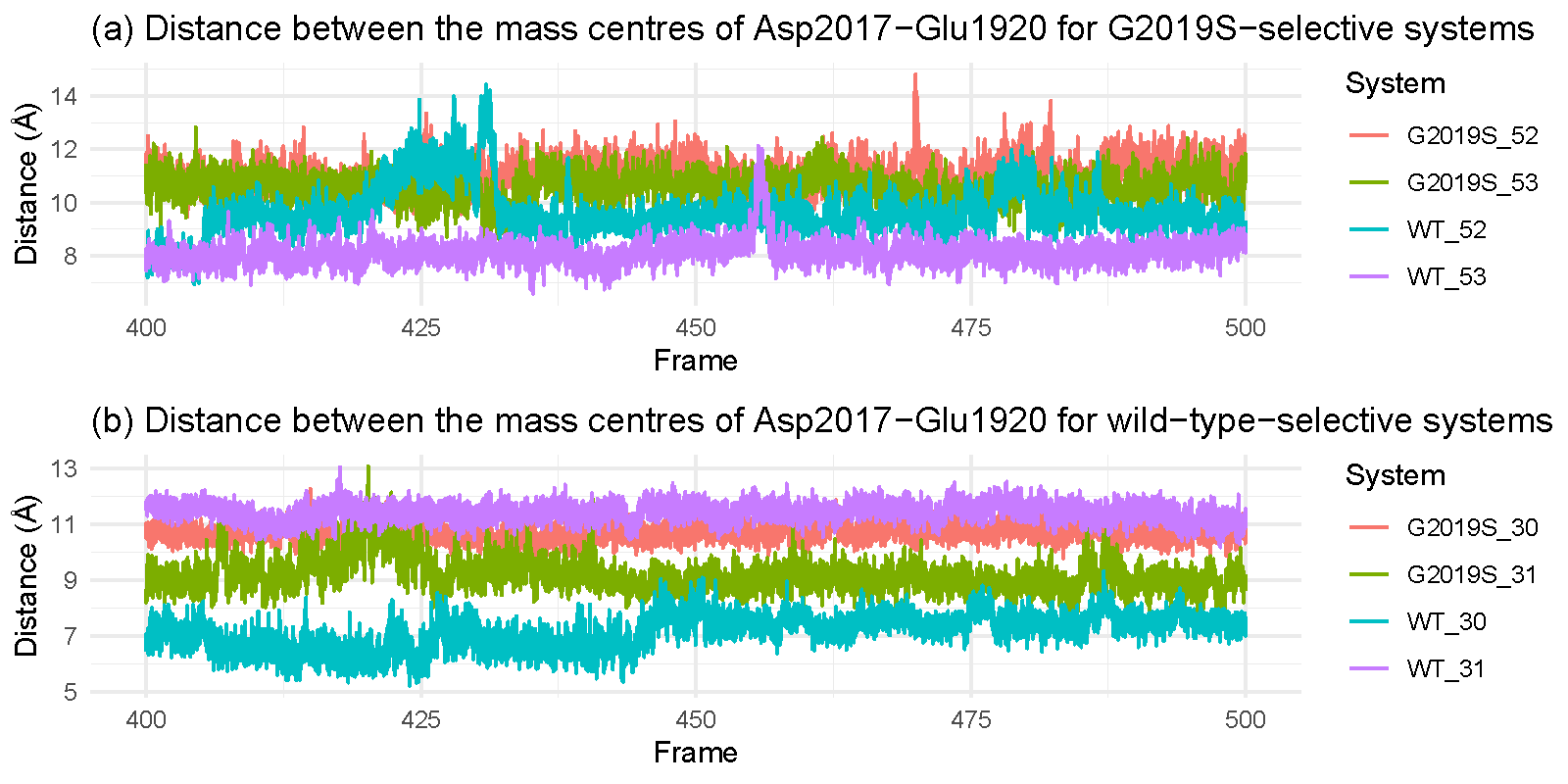


**Fig. S5** Distance between the mass centres of Asp2017-Glu1920 in the last 100 ns for: (a) G2019S-selective and (b) wild-type-selective inhibitors.


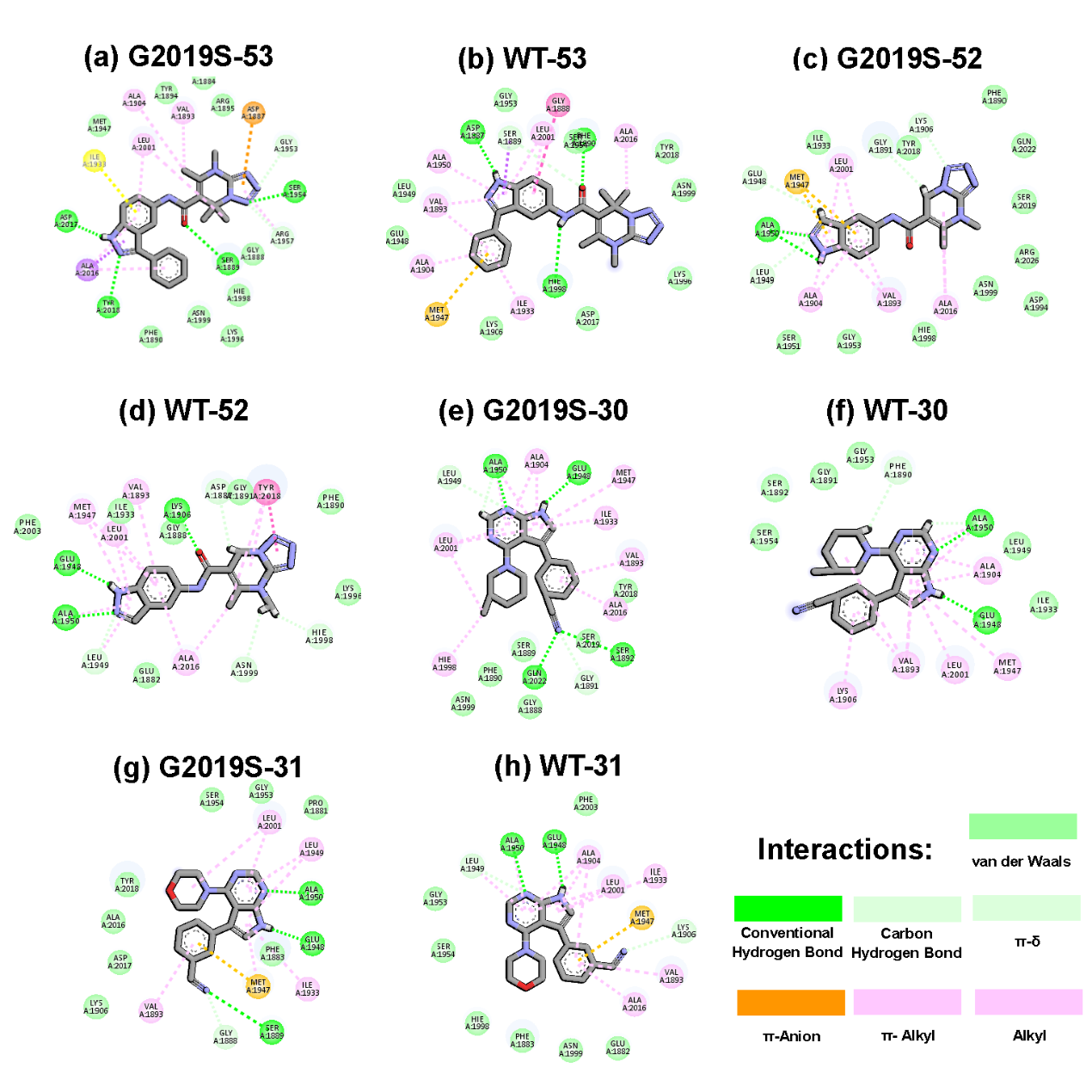


**Fig. S6** 2D Binding patterns in the ground minima for all systems.

**Table S3** Per residue decomposition free energy for the significant residues (kcal/mol).

| Residue | GS53 | WT53 | GS52 | WT52 | GS30 | WT30 | GS31 | WT31 |
| --- | --- | --- | --- | --- | --- | --- | --- | --- |
| Ala1904 | -0.32740 | -1.12059 | -0.70162 | -0.20871 | -0.77398 | -0.93796 | -0.14611 | -1.18889 |
| Val1905 | -0.14604 | -0.12564 | -0.03181 | -0.04829 | -0.10242 | -0.10255 | -0.08764 | -0.22337 |
| Lys1906 | 0.10504 | -0.50386 | -0.32139 | 0.14578 | 0.10022 | -0.47681 | 0.04619 | -0.74341 |
| Ile1933 | -0.79314 | -0.84972 | -0.84399 | -1.03599 | -0.85940 | -0.51958 | -1.23877 | -0.83841 |
| Glu1948 | 0.04861 | -0.00033 | 0.14872 | -1.83020 | -2.04777 | -2.11219 | -2.22802 | -1.94345 |
| Leu1949 | -0.24722 | -0.94700 | -1.56298 | -1.58346 | -1.86780 | -2.07832 | -2.30274 | -2.15939 |
| Ala1950 | -0.15865 | -0.88074 | -1.32128 | -1.14092 | -1.60167 | -1.80459 | -1.69397 | -1.56600 |
| Ser1951 | 0.02280 | -0.01067 | 0.03792 | 0.04297 | 0.04114 | -0.02313 | 0.05050 | 0.02002 |
| Ser1954 | -1.27977 | -0.52111 | -0.08673 | -0.06532 | -0.13387 | -0.45486 | -0.25428 | -0.47399 |
| Arg1957 | -1.23539 | -0.01526 | 0.07632 | 0.06681 | 0.04326 | 0.07619 | 0.01383 | 0.07868 |
| Hie1998 | -0.22233 | -2.34816 | 0.03370 | -0.07947 | -0.28969 | 0.00753 | 0.03057 | -0.31036 |
| Asn1999 | -1.21684 | -2.01861 | -0.48806 | -0.82727 | -0.41302 | 0.01349 | -0.00964 | -0.36355 |
| Leu2001 | -1.83648 | -2.32102 | -2.13245 | -1.57104 | -2.17777 | -1.98105 | -1.91213 | -2.32606 |
| Ala2016 | -1.45390 | -0.69248 | -0.97422 | -0.38517 | -0.36862 | -0.12062 | -0.47181 | -1.06318 |
| Asp2017 | -0.50112 | 1.04268 | -0.10015 | 0.12294 | -0.23760 | 0.38515 | 0.90114 | -0.01659 |
| Tyr2018 | -1.52895 | -0.99017 | -0.73777 | -3.38217 | -1.23364 | -0.06109 | -1.59560 | -0.10561 |
| Gln2022 | 0.00729 | 0.00176 | -0.73426 | -0.00811 | -0.80140 | 0.01600 | -0.03191 | 0.00834 |


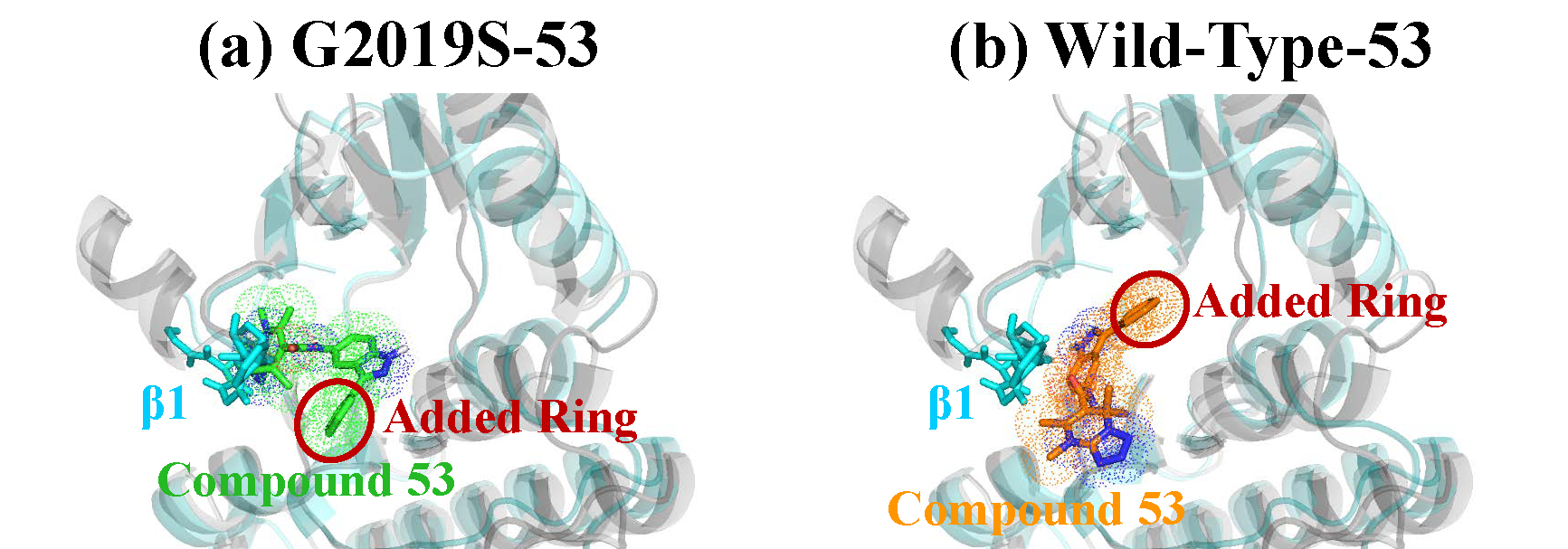


**Fig S7** Distinct binding orientations of Compound 53 in aligned G2019S (grey) and wild-type (cyan) LRRK2 kinases. The ligand from the G2019S system is shown in green, while the ligand from the wild-type system is shown in orange.


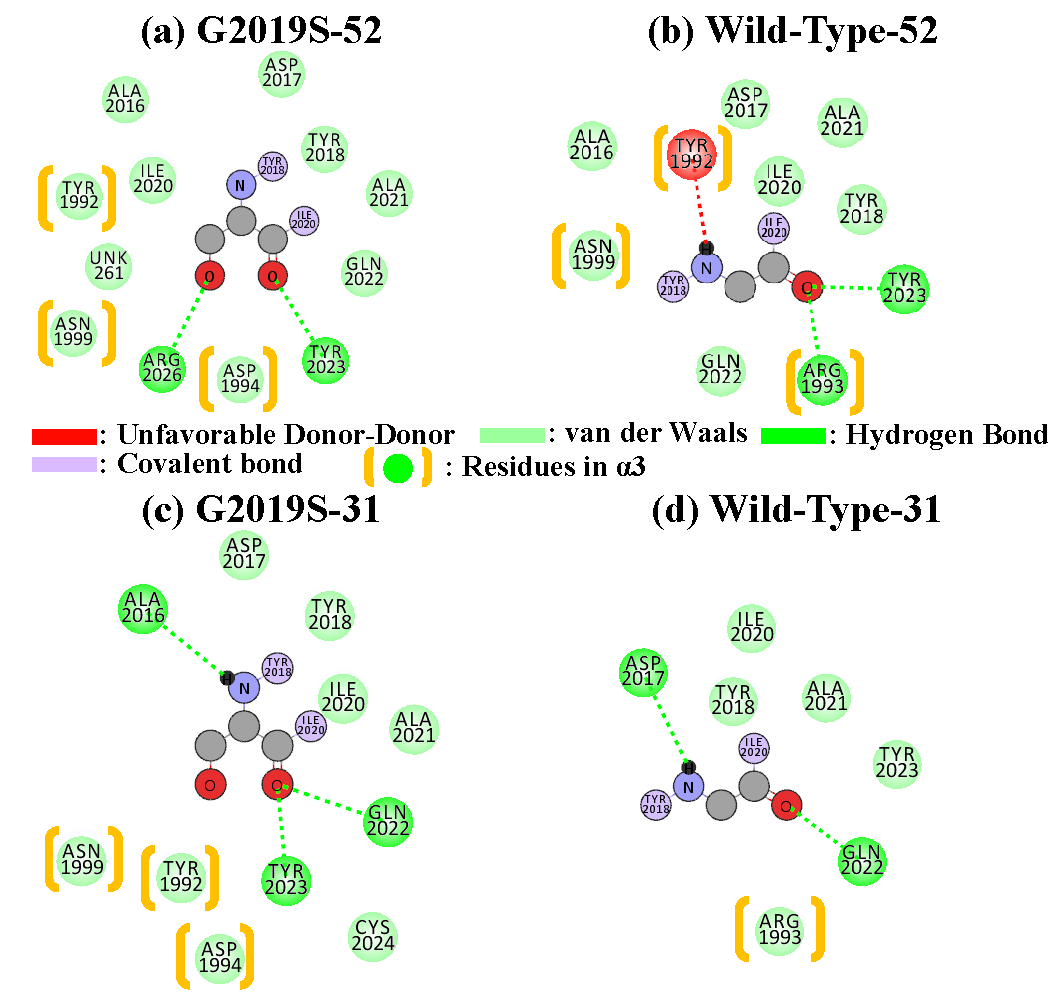


**Fig S8** Interactions between residue 2019 (Gly in wild-type or Ser in G2019S) and surrounding residues in the Compound 31 and Compound 52 systems.
